# 3-(4-Methoxy­phen­yl)-1-(2-pyrrol­yl)prop-2-en-1-one

**DOI:** 10.1107/S160053680802864X

**Published:** 2008-09-13

**Authors:** Dong-Qing Li

**Affiliations:** a Department of Chemistry and Biology, Yulin Normal University, Guangxi 537000, People’s Republic of China

## Abstract

The title mol­ecule, C_14_H_13_NO_2_, is almost flat with a dihedral angle of 8.0 (1)° between the pyrrole and benzene rings. The central C_3_O ketone unit has an *s-cis* conformation and is also coplanar with a torsion angle of −0.6 (3) °. An intra­molecular C—H⋯O hydrogen bond generates an *S*(5) ring motif. In addition, the meth­oxy group is coplanar with the attached benzene ring. In the crystal structure, neighboring mol­ecules are paired through N—H⋯O hydrogen bonds into centrosymmetric dimers with an *R* ^2^_2_(10) motif.

## Related literature

For the pharmaceutical and biological properties of chalcones, see: Lin *et al.* (2002 ▶); Modzelewska *et al.* (2006 ▶); Opletalova (2000 ▶); Opletalova & Sedivy (1999 ▶); Sogawa *et al.* (1994 ▶). For chalcones as non-linear optical materials, see: Agrinskaya *et al.* (1999 ▶); Indira *et al.* (2002 ▶). For related structures, see: Bukhari *et al.* (2008 ▶); Fun *et al.* (2008 ▶); Gong, *et al.* (2008 ▶).
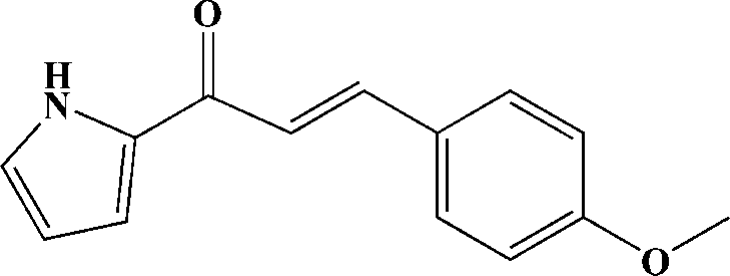


## Experimental

### 

#### Crystal data

C_14_H_13_NO_2_
*M* *_r_* = 227.25
Monoclinic, 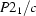

*a* = 5.0815 (7) Å
*b* = 17.172 (3) Å
*c* = 13.973 (2) Å
β = 97.878 (3)°
*V* = 1207.8 (3) Å^3^
*Z* = 4
Mo *K*α radiation
μ = 0.08 mm^−1^
*T* = 293 (2) K
0.40 × 0.24 × 0.20 mm

#### Data collection

Bruker SMART APEX area-detector diffractometer
Absorption correction: multi-scan (*SADABS*; Sheldrick, 1996 ▶) *T*_min_ = 0.967, *T*_max_ = 0.988
5842 measured reflections
2369 independent reflections
1809 reflections with *I* > 2σ(*I*)
*R*_int_ = 0.019

#### Refinement

*R*[*F* ^2^ > 2σ(*F* ^2^)] = 0.047
*wR*(*F* ^2^) = 0.124
*S* = 1.05
2369 reflections
155 parameters
H-atom parameters constrained
Δρ_max_ = 0.13 e Å^−3^
Δρ_min_ = −0.13 e Å^−3^

### 

Data collection: *SMART* (Bruker, 2002 ▶); cell refinement: *SAINT* (Bruker, 2002 ▶); data reduction: *SAINT*; program(s) used to solve structure: *SHELXS97* (Sheldrick, 2008 ▶); program(s) used to refine structure: *SHELXL97* (Sheldrick, 2008 ▶); molecular graphics: *SHELXTL* (Sheldrick, 2008 ▶); software used to prepare material for publication: *SHELXTL*.

## Supplementary Material

Crystal structure: contains datablocks I, global. DOI: 10.1107/S160053680802864X/cs2091sup1.cif

Structure factors: contains datablocks I. DOI: 10.1107/S160053680802864X/cs2091Isup2.hkl

Additional supplementary materials:  crystallographic information; 3D view; checkCIF report

## Figures and Tables

**Table 1:** Hydrogen-bond geometry (Å, °)

*D*—H⋯*A*	*D*—H	H⋯*A*	*D*⋯*A*	*D*—H⋯*A*
C7—H7⋯O1	0.93	2.52	2.838 (2)	100
N1—H1⋯O1^i^	0.86	2.03	2.8314 (17)	155

Symmetry code: (i) 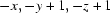.

**Fig. 1.**
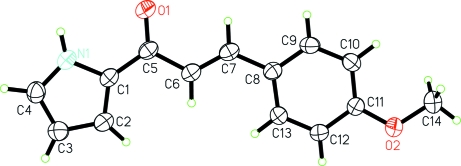
The molecular structure of the title compound with displacement ellipsoids drawn at the 30% probability level and H atoms as spheres of arbitrary radius.

**Fig. 2.**
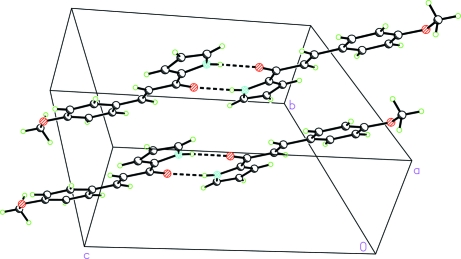
Packing diagram of the title structure showing the N—H···.O hydrogen bonding interactions.

## supplementary crystallographic information

### Comment

Chalcone derivatives have recently attracted extensive interest due to possessing a wide variety of pharmaceutical (Lin *et al.*, 2002; Modzelewska *et al.*, 2006; Sogawa *et al.*, 1994) and biological properties (Opletalova, 2000; Opletalova & Sedivy, 1999). Some substituted chalcones also exhibit the potential applications as non-linear optical materials (Agrinskaya *et al.*, 1999; Indira *et al.*, 2002). Considering the importance of these types of compounds, a new chalcone compound was synthesized and its crystal structure is reported here.

The molecular structure of the title molecule (Fig. 1) is almost planar as indicated by a dihedral angle of 8.0 (1) ° between the pyrrole and benzene rings. The central O1/C5/C6/C7 ketone motif exhibits an *s-cis* conformation as usual in other related chalcone derivatives (Bukhari *et al.*, 2008; Fun *et al.*, 2008; Gong, *et al.*, 2008;) and also coplanar with a torsion angle of -0.6 (3) °, meanwhile, O1 atom acts as an acceptor and is involved in an intramolecular C—H···O hydrogen bond (Table 1) to generate an S(5) ring motif. In the crystal packing, the compound can be stabilized by intermolecular N—H···O hydrogen bonds with –NH groups as donors to form centrosymmetric dimers with an *R*^2^_2_(10) motif as shown in Fig. 2.

### Experimental

The title compound was synthesized by the condensation of 2-acetylpyrrole (1.09 g, 10.0 mmol) and 4-methoxybenzaldehyde (1.06 g, 5.0 mmol) in methanol (30 ml) and ammonia (25%, 25 ml) in the presence of sodium hydroxide (0.56 g, 10 mmol). After refluxed at 358 K for 8 h, the contents of the flask were cooled to give a yellow crude precipitate which was separated by filtration, washed with water and iced ethanol. Recrystallization from ethanol afforded yellow prism-like crystals. Yield: 0.85 g (74.8%).

### Refinement

All H-atoms were positioned geometrically and refined using a riding model with d(C—H) = 0.93 Å, *U*_iso_=1.2*U*_eq_ (C) for aromatic and ethylene; 0.96 Å, *U*_iso_= 1.5*U*_eq_ (C) for CH_3_ atoms, and d(N—H) = 0.86 Å, *U*_iso_=1.2*U*_eq_ (N) for pyrrole nitrogen atom.

### Crystal data

**Table:**

C_14_H_13_NO_2_	*F*(000) = 480
*M**_r_* = 227.25	*D*_x_ = 1.250 Mg m^−^^3^
Monoclinic, *P*2_1_/*c*	Mo *K*α radiation, λ = 0.71073 Å
Hall symbol: -P 2ybc	Cell parameters from 1716 reflections
*a* = 5.0815 (7) Å	θ = 2.4–25.8°
*b* = 17.172 (3) Å	µ = 0.08 mm^−^^1^
*c* = 13.973 (2) Å	*T* = 293 K
β = 97.878 (3)°	Prism, yellow
*V* = 1207.8 (3) Å^3^	0.40 × 0.24 × 0.20 mm
*Z* = 4	

### Data collection

**Table:**

Bruker APEX area-detector diffractometer	2369 independent reflections
Radiation source: fine-focus sealed tube	1809 reflections with *I* > 2σ(*I*)
graphite	*R*_int_ = 0.020
φ and ω scans	θ_max_ = 26.0°, θ_min_ = 1.9°
Absorption correction: multi-scan (SADABS; Sheldrick, 1996)	*h* = −6→5
*T*_min_ = 0.967, *T*_max_ = 0.988	*k* = −20→21
5842 measured reflections	*l* = −14→17

### Refinement

**Table:**

Refinement on *F*^2^	Primary atom site location: structure-invariant direct methods
Least-squares matrix: full	Secondary atom site location: difference Fourier map
*R*[*F*^2^ > 2σ(*F*^2^)] = 0.047	Hydrogen site location: inferred from neighbouring sites
*wR*(*F*^2^) = 0.124	H-atom parameters constrained
*S* = 1.05	*w* = 1/[σ^2^(*F*_o_^2^) + (0.0557*P*)^2^ + 0.1497*P*] where *P* = (*F*_o_^2^ + 2*F*_c_^2^)/3
2369 reflections	(Δ/σ)_max_ < 0.001
155 parameters	Δρ_max_ = 0.13 e Å^−^^3^
0 restraints	Δρ_min_ = −0.13 e Å^−^^3^

### Fractional atomic coordinates and isotropic or equivalent isotropic displacement parameters (Å^2^)

**Table:**

	*x*	*y*	*z*	*U*_iso_*/*U*_eq_	
N1	−0.2188 (3)	0.56328 (8)	0.38761 (9)	0.0567 (4)	
H1	−0.1976	0.5437	0.4448	0.068*	
O1	0.1957 (3)	0.45435 (7)	0.40994 (8)	0.0707 (4)	
O2	0.9171 (3)	0.29580 (8)	−0.06480 (9)	0.0769 (4)	
C1	−0.0745 (3)	0.54326 (9)	0.31541 (10)	0.0520 (4)	
C2	−0.1690 (4)	0.58892 (10)	0.23690 (12)	0.0634 (5)	
H2	−0.1082	0.5885	0.1771	0.076*	
C3	−0.3705 (4)	0.63554 (11)	0.26280 (13)	0.0707 (5)	
H3	−0.4688	0.6721	0.2238	0.085*	
C4	−0.3978 (3)	0.61792 (11)	0.35599 (12)	0.0634 (5)	
H4	−0.5202	0.6401	0.3916	0.076*	
C5	0.1294 (3)	0.48406 (9)	0.32984 (11)	0.0535 (4)	
C6	0.2491 (3)	0.45958 (10)	0.24510 (11)	0.0570 (4)	
H6	0.1913	0.4834	0.1862	0.068*	
C7	0.4347 (3)	0.40549 (10)	0.24796 (11)	0.0556 (4)	
H7	0.4909	0.3835	0.3081	0.067*	
C8	0.5616 (3)	0.37631 (9)	0.16757 (11)	0.0517 (4)	
C9	0.7403 (3)	0.31532 (10)	0.17995 (11)	0.0578 (4)	
H9	0.7789	0.2930	0.2409	0.069*	
C10	0.8645 (3)	0.28594 (10)	0.10527 (12)	0.0586 (4)	
H10	0.9827	0.2445	0.1160	0.070*	
C11	0.8105 (3)	0.31894 (10)	0.01503 (11)	0.0563 (4)	
C12	0.6337 (4)	0.38052 (11)	0.00062 (12)	0.0714 (5)	
H12	0.5978	0.4033	−0.0602	0.086*	
C13	0.5110 (4)	0.40830 (11)	0.07518 (12)	0.0669 (5)	
H13	0.3915	0.4494	0.0639	0.080*	
C14	1.0982 (4)	0.23244 (12)	−0.05485 (15)	0.0873 (6)	
H14A	1.2445	0.2445	−0.0060	0.131*	
H14B	1.1631	0.2235	−0.1153	0.131*	
H14C	1.0094	0.1866	−0.0365	0.131*	

### Atomic displacement parameters (Å^2^)

**Table:**

	*U*^11^	*U*^22^	*U*^33^	*U*^12^	*U*^13^	*U*^23^
N1	0.0611 (8)	0.0626 (9)	0.0479 (7)	−0.0046 (7)	0.0129 (6)	−0.0043 (6)
O1	0.0880 (9)	0.0760 (8)	0.0520 (7)	0.0121 (7)	0.0233 (6)	0.0115 (6)
O2	0.0977 (9)	0.0780 (9)	0.0577 (7)	0.0310 (7)	0.0207 (7)	0.0018 (6)
C1	0.0533 (9)	0.0579 (10)	0.0459 (8)	−0.0089 (7)	0.0107 (7)	−0.0036 (7)
C2	0.0698 (11)	0.0717 (12)	0.0499 (9)	0.0006 (9)	0.0120 (8)	0.0007 (8)
C3	0.0771 (12)	0.0731 (12)	0.0605 (11)	0.0098 (10)	0.0042 (9)	0.0001 (9)
C4	0.0606 (10)	0.0670 (11)	0.0630 (10)	0.0017 (9)	0.0098 (8)	−0.0132 (9)
C5	0.0581 (9)	0.0570 (10)	0.0468 (8)	−0.0110 (8)	0.0120 (7)	0.0004 (7)
C6	0.0610 (10)	0.0631 (10)	0.0480 (8)	−0.0034 (8)	0.0112 (7)	0.0020 (7)
C7	0.0607 (10)	0.0594 (10)	0.0473 (8)	−0.0088 (8)	0.0097 (7)	0.0029 (7)
C8	0.0562 (9)	0.0501 (9)	0.0492 (8)	−0.0053 (7)	0.0084 (7)	0.0006 (7)
C9	0.0609 (10)	0.0617 (11)	0.0499 (9)	0.0015 (8)	0.0049 (8)	0.0112 (7)
C10	0.0583 (10)	0.0567 (10)	0.0604 (10)	0.0089 (8)	0.0062 (8)	0.0056 (8)
C11	0.0636 (10)	0.0549 (10)	0.0507 (9)	0.0046 (8)	0.0096 (8)	−0.0014 (7)
C12	0.0972 (14)	0.0701 (12)	0.0473 (9)	0.0254 (11)	0.0115 (9)	0.0082 (8)
C13	0.0855 (13)	0.0608 (11)	0.0553 (10)	0.0232 (9)	0.0137 (9)	0.0050 (8)
C14	0.1013 (15)	0.0851 (14)	0.0779 (13)	0.0345 (13)	0.0212 (11)	−0.0047 (11)

### Geometric parameters (Å, °)

**Table:**

N1—C4	1.339 (2)	C7—C8	1.459 (2)
N1—C1	1.3699 (19)	C7—H7	0.9300
N1—H1	0.8600	C8—C9	1.382 (2)
O1—C5	1.2342 (18)	C8—C13	1.394 (2)
O2—C11	1.3644 (19)	C9—C10	1.386 (2)
O2—C14	1.419 (2)	C9—H9	0.9300
C1—C2	1.380 (2)	C10—C11	1.375 (2)
C1—C5	1.446 (2)	C10—H10	0.9300
C2—C3	1.386 (2)	C11—C12	1.384 (2)
C2—H2	0.9300	C12—C13	1.371 (2)
C3—C4	1.362 (2)	C12—H12	0.9300
C3—H3	0.9300	C13—H13	0.9300
C4—H4	0.9300	C14—H14A	0.9600
C5—C6	1.465 (2)	C14—H14B	0.9600
C6—C7	1.320 (2)	C14—H14C	0.9600
C6—H6	0.9300		
			
C4—N1—C1	109.93 (14)	C9—C8—C13	116.70 (15)
C4—N1—H1	125.0	C9—C8—C7	121.10 (14)
C1—N1—H1	125.0	C13—C8—C7	122.20 (15)
C11—O2—C14	117.91 (14)	C8—C9—C10	122.74 (15)
N1—C1—C2	106.24 (15)	C8—C9—H9	118.6
N1—C1—C5	121.30 (14)	C10—C9—H9	118.6
C2—C1—C5	132.46 (15)	C11—C10—C9	119.07 (15)
C1—C2—C3	108.04 (15)	C11—C10—H10	120.5
C1—C2—H2	126.0	C9—C10—H10	120.5
C3—C2—H2	126.0	O2—C11—C10	125.29 (15)
C4—C3—C2	107.26 (17)	O2—C11—C12	115.23 (14)
C4—C3—H3	126.4	C10—C11—C12	119.48 (15)
C2—C3—H3	126.4	C13—C12—C11	120.59 (16)
N1—C4—C3	108.52 (15)	C13—C12—H12	119.7
N1—C4—H4	125.7	C11—C12—H12	119.7
C3—C4—H4	125.7	C12—C13—C8	121.42 (16)
O1—C5—C1	121.24 (14)	C12—C13—H13	119.3
O1—C5—C6	121.48 (16)	C8—C13—H13	119.3
C1—C5—C6	117.27 (14)	O2—C14—H14A	109.5
C7—C6—C5	123.47 (15)	O2—C14—H14B	109.5
C7—C6—H6	118.3	H14A—C14—H14B	109.5
C5—C6—H6	118.3	O2—C14—H14C	109.5
C6—C7—C8	127.43 (15)	H14A—C14—H14C	109.5
C6—C7—H7	116.3	H14B—C14—H14C	109.5
C8—C7—H7	116.3		
			
C4—N1—C1—C2	0.86 (18)	C6—C7—C8—C9	−175.31 (17)
C4—N1—C1—C5	−178.71 (14)	C6—C7—C8—C13	5.0 (3)
N1—C1—C2—C3	−0.38 (19)	C13—C8—C9—C10	−0.4 (3)
C5—C1—C2—C3	179.11 (17)	C7—C8—C9—C10	179.89 (15)
C1—C2—C3—C4	−0.2 (2)	C8—C9—C10—C11	0.6 (3)
C1—N1—C4—C3	−1.01 (19)	C14—O2—C11—C10	0.2 (3)
C2—C3—C4—N1	0.7 (2)	C14—O2—C11—C12	−179.46 (18)
N1—C1—C5—O1	−6.2 (2)	C9—C10—C11—O2	−179.72 (16)
C2—C1—C5—O1	174.32 (17)	C9—C10—C11—C12	−0.1 (3)
N1—C1—C5—C6	172.35 (14)	O2—C11—C12—C13	179.16 (17)
C2—C1—C5—C6	−7.1 (3)	C10—C11—C12—C13	−0.5 (3)
O1—C5—C6—C7	−0.6 (3)	C11—C12—C13—C8	0.6 (3)
C1—C5—C6—C7	−179.16 (15)	C9—C8—C13—C12	−0.2 (3)
C5—C6—C7—C8	178.75 (15)	C7—C8—C13—C12	179.49 (16)

### Hydrogen-bond geometry (Å, °)

**Table:**

*D*—H···*A*	*D*—H	H···*A*	*D*···*A*	*D*—H···*A*
C7—H7···O1	0.93	2.52	2.838 (2)	100.
N1—H1···O1^i^	0.86	2.03	2.8314 (17)	155.

Symmetry codes: (i) −*x*, −*y*+1, −*z*+1.

## References

[bb1] Agrinskaya, N. V., Lukoshkin, V. A., Kudryavtsev, V. V., Nosova, G. I., Solovskaya, N. A. & Yakimanski, A. V. (1999). *Phys. Solid State*, **41**, 1914–1917.

[bb2] Bruker (2002). *SMART* and *SAINT* Bruker AXS Inc., Madison, Wisconsin, USA.

[bb3] Bukhari, M. H., Siddiqui, H. L., Tahir, M. N., Chaudhary, M. A. & Iqbal, A. (2008). *Acta Cryst.* E**64**, o867–o868. 10.1107/S1600536808010362 PMC2961151 21202354

[bb4] Fun, H.-K., Chantrapromma, S., Patil, P. S., Karthikeyan, M. S. & Dharmaprakash, S. M. (2008). *Acta Cryst.* E**64**, o956–o957. 10.1107/S1600536808012178 PMC2961420 21202688

[bb5] Gong, Z.-Q., Liu, G.-S. & Xia, H.-Y. (2008). *Acta Cryst.* E**64**, o151. 10.1107/S1600536807063489 PMC2915219 21200716

[bb6] Indira, J., Prakash Karat, P. & Sarojini, B. K. (2002). *J. Cryst. Growth*, **242**, 209–214.

[bb7] Lin, Y. M., Zhou, Y., Flavin, M. T., Zhou, L. M., Nie, W. & Chen, F. C. (2002). *Bioorg. Med. Chem.* **10**, 2795–2802. 10.1016/s0968-0896(02)00094-9 12057669

[bb8] Modzelewska, A., Catherine Petit, C., Achanta, G., Davidson, N. E., Huang, P. & Khan, S. R. (2006). *Bioorg. Med. Chem.* **14**, 3491–3495. 10.1016/j.bmc.2006.01.003 16434201

[bb9] Opletalova, V. (2000). *Ceska Slov. Farm.* **49**, 278–284. 11367546

[bb10] Opletalova, V. & Sedivy, D. (1999). *Ceska Slov. Farm.* **48**, 252–255. 10748740

[bb11] Sheldrick, G. M. (1996). *SADABS* University of Göttingen, Germany.

[bb12] Sheldrick, G. M. (2008). *Acta Cryst.* A**64**, 112–122. 10.1107/S0108767307043930 18156677

[bb13] Sogawa, S., Nihro, Y., Ueda, H., Miki, T., Matsumoto, H. & Satoh, T. (1994). *Biol. Pharm. Bull.* **17**, 251–256. 10.1248/bpb.17.251 8205123

